# Predictors of institutionalization in users of day care facilities with mild cognitive impairment to moderate dementia

**DOI:** 10.1186/s12913-021-07017-8

**Published:** 2021-09-24

**Authors:** Klara Spiegl, Katharina Luttenberger, Elmar Graessel, Linda Becker, Jennifer Scheel, Anna Pendergrass

**Affiliations:** 1 Department of Medical Psychology and Medical Sociology, University Clinic for Psychiatry and Psychotherapy, Erlangen University Hospital, Friedrich-Alexander University Erlangen Nuremberg, Erlangen, Germany, Schwabachanlage 6, 91054 Erlangen, Germany; 2 Department of Health Psychology, Friedrich-Alexander University Erlangen-Nuernberg, Nägelsbachstr. 49a, 91052 Erlangen, Germany

**Keywords:** Dementia patient, Mild cognitive impairment, MCI, Institutionalization, Informal caregivers

## Abstract

**Background:**

Most people with dementia wish to remain at home for as long as possible. Therefore, it is important to know the predictors of institutionalization, especially those that can be influenced. The aim of the present study is to identify predictors of the institutionalization of people with mild cognitive impairment (MCI) to moderate dementia who attend day care facilities (DCFs) throughout Germany.

**Methods:**

This study is a secondary analysis of longitudinal data from 371 dyads comprising a cognitively impaired care receiver (CR) and a caregiver (CG). The data were collected in DCFs and via telephone interviews at three measurement points. To investigate the extent to which 16 variables could predict the institutionalization of the CRs between the 6- and 12-month follow-up, in the first step bivariate Cox regressions were calculated. In the second step, significant predictors were included in a model using multivariate Cox regression.

**Results:**

Between the 6- and 12-month evaluations, 39 CRs moved into an institution. The risk of institutionalization of people with MCI to moderate dementia attending a DCF increased significantly (*p* < .05) when the CRs showed more neuropsychiatric symptoms (Hazard ratio (HR) = 1.237), when the CRs and their CGs did not live together in the same house (HR = 2.560), or when the care level of the CRs is low (HR = 2.241).

**Conclusions:**

Neuropsychiatric symptoms could be a possible starting point for therapeutic interventions that are designed to delay or prevent institutionalization. CG who do not live with their CR in the same house and CG who care for a CR with impairment in performing daily routine tasks care are particularly likely to make the decision to institutionalize the CR. For this group, advice and support are particularly important.

**Trail registration:**

ISRCTN16412551.

## Background

People with dementia in most cases hope that they can stay at home for as long as possible [1]. For them, institutionalization means losing their long-term home, their own independence, and their familiar social environment [2]. In addition, their physical and mental health tend to suffer, and this is associated with a higher risk of morbidity and mortality [3]. Besides the care receivers (CRs), the institutionalization also affects the family caregivers (CGs) directly. Although CGs tend to note a reduction in everyday physical and emotional burdens immediately after their CR is institutionalized [4], CGs depressive symptoms and anxiety usually remain as high as before [5]. The institutionalization also usually brings financial problems because it is the most expensive option for the family. Although long-term care insurance can ease the burden, a self-share (up to 2000 euros per month in Germany) has to be borne by those affected [2].

In Germany, it is assumed that currently about 1.5 to 3.7 million people are affected by MCI [6] and people with MCI have a 2 to 4.6 times higher annual rate of conversion to dementia [7]. As the German population continues to age, the number of people with MCI and the number of people with MCI who develop dementia will increase, which will pose major challenges for the care of this group [6]. Day care facilities (DCFs) are a support offer that reduces the burden of care for CGs [8]. Based on the increasing number of people in need of care, this offer is becoming increasingly important. Many of the DCFs are specialized on people with dementia as “gerontopsychiatric DCFs” [9]. To the best of our knowledge, there have not been any studies searching for the predictors of institutionalization of people with mild cognitive impairment to moderate dementia who visit a DCF.

Many studies with different target groups have focused on predictors of institutionalization. Eska and colleagues for example focused in their study particular on patients with mild to moderate dementia who were cared for at home [2]. In line with other research results, they found that institutionalization was more likely (a) for older patients [2], (b) for older CGs [2, 10–12], (c) when CGs and CRs did not live together [2, 13], (d) when CGs had a high educational level [2], (e) when the subjective burden on CGs was high [2, 14], and (f) when community health services were used a lot [2, 15, 16].

These six predictors are not easy to influence. However, to prevent institutionalization it is especially important to focus on predictors that can be influenced or even prevented through interventions, for example, cognitive skills, everyday practical skills, and neuropsychiatric symptoms [17–20]. These factors also strongly influence the required amount of care that is reflected in the care level in Germany [2]. Heinen and colleagues were able to show that the risk of institutionalization in people with dementia increased in general with an increase in the care level [21]. The present study focused on one specific subgroup of people, which has not been a major focus of research so far. Every CR had MCI, mild dementia, or moderate dementia and regularly visited a DCF. The focus is thus on people with MCI and the earlier stages of dementia. For this group, we wanted to develop a model for predicting institutionalization. Thereby, our interest was in determining which variables across a 6-month time period would be significant predictors of institutionalization in a multivariate context.

## Methods

### Research design

**Fig. 1:**
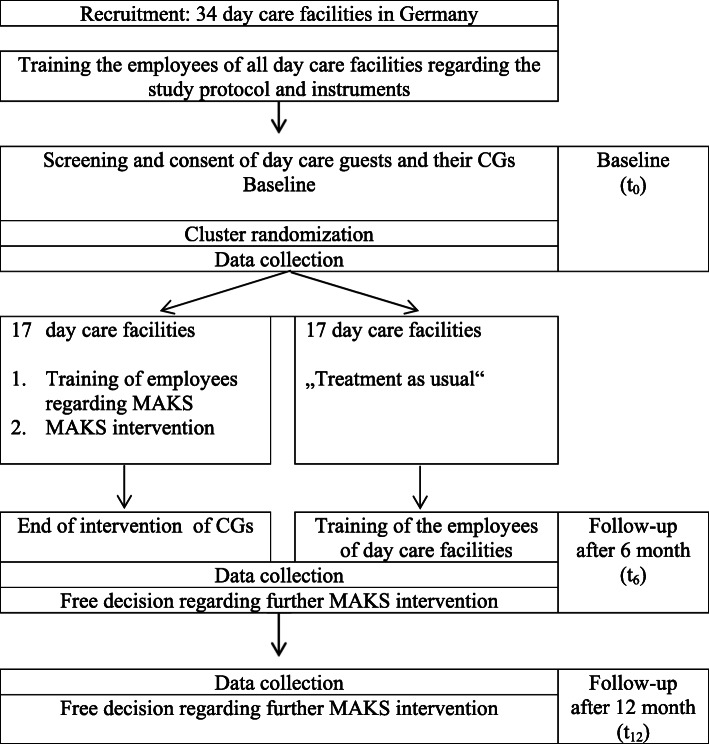
Flow chart (short version)

We used data from the German day-care study, a cluster randomized, controlled, single-blind follow-up study with a 6-month intervention phase in the waitlist control group design ([22]; for main findings see: [20]). In the intervention group, MAKS therapy was carried out for 6 months, for which the employees of the DCFs had previously been trained. The control group received treatment as usual. The data were collected at three different points: at the beginning of the study (t0); after the intervention phase (t6), and after 12 months (t12). The data were also collected at different levels: The DCF employees collected data from the CRs, and the CGs were interviewed on the telephone (see Fig. 1) [22]. The elements of the study were approved by the ethics committee of the Medical Faculty of the Friedrich-Alexander-University Erlangen-Nuremberg, and the study design was published in advance and carried out as described [22].

### Criteria for inclusion and exclusion

Two-stage screening excluded those who had at least one of the following: blindness or deafness, poor communication skills, more than one stroke, severe depression, schizophrenia, an addiction disorder, no CG, already existing concrete plans for institutionalization, or presence in the DCF less often than once a week. In a second step, neuropsychological tests (Mini-Mental State Examination (MMSE) and Montreal Cognitive Assessment (MoCa)) were used to determine whether MCI or mild to moderate dementia was present among the visitors of the DCF [22]. Thus, a sample of 453 participants who had had MCI, mild dementia, or moderate dementia from 34 different DCFs from all over Germany was recruited at the beginning of the study.

### Participants

In our analysis, all dyads of CRs and CGs for which we had valid data on care situation (e.g. institutionalization, death, DCF attendance) were included. Of the 453 eligible dyads at t0, 35 participants had already transferred to the nursing home before the first measurement point (t6) and 47 had died before the second measurement point (t12). This results in a sample of 371 dyads. Between t6 and t12, 39 CRs moved into a nursing home. More information about the sample can be found in Table 1.

**Table 1:** Descriptive Statistics

Second Measurement	Institutionalization *(n = 39, 100%)*	No Institutionalization *(n = 332, 100%)*	Test for group differences *p*	Total (*N = 371, 100%*)
Caregiver				
Age, mean (*SD*)	58.46 (10.2)	59.55 (11.6)	.576 ^a^	59.44 (11.5)
Women, number (*%*)	29 (74.4)	244 (73.5)	.908 ^b^	273 (73.6)
Education in years, mean (*SD*)	11.36 (3.1)	10.89 (2.9)	.337 ^a^	10.94 (2.9)
Employed, number (%)	23 (59.0)	175 (52.7)	.458 ^b^	198 (53.4)
Relationship to CR, number (%)			.069 ^b^*	
Married/long-term relationship	5 (12.8)	94 (28.3)		99 (26.7)
Daughter/son	26 (66.7)	198 (59.6)		224 (60.4)
Daughter−/son-in-law	3 (7.7)	21 (6.3)		24 (6.5)
Other relatives	4 (10.3)	17 (5.1)		21 (5.7)
Friend/neighbor	1 (2.6)	2 (.6)		3 (.8)
**Living together with CR, number (%)**	16 (41.0)	213 (64.2)	**.005**^**b**^	229 (61.7)
Subjective burden (BSFC-s), mean (*SD*)	13.36 (8.0)	11.84 (7.7)	.250 ^a^	12.00 (7.8)
Telephone support intervention, mean (SD)	15 (38.8)	103 (31.0)	.345 ^a^	118 (31.8)
Care receiver				
Age, mean (*SD*)	83.46 (7.4)	81.11 (7.7)	.070 ^a^	81.36 (7.7)
Women, number (*%*)	25 (64.1)	206 (62.0)	.802 ^b^	231 (62.3)
Living in relationship, number (*%*)	13 (33.3)	134 (40.4)	.396 ^b^	147 (39.6)
MAKS therapy received, number (*%*)	27 (69.2)	187 (56.3)	.123 ^b^	214 (57.7)
Antidementive drug, number (%)	15 (38.5)	103 (31.0)	.345 ^b^	118 (31.8)
Use of community health services, mean (*SD*)	2.05 (1.4)	1.80 (1.3)	.269 ^a^	1.83 (1.3)
**Neuropsychiatric symptoms (NPI-Q), mean (*SD*)**	6.56 (2.6)	5.15 (2.6)	**.002**^**a**^	5.30 (2.6)
Everyday practical skills (ETAM), mean (*SD*)	16.67 (6.8)	17.72 (7.5)	.409 ^a^	17.61 (7.4)
Cognitive restrictions (MMSE), mean (*SD*)	18.39 (5.4)	19.32 (6.0)	.354 ^a^	19.22 (5.9)
**Care level, number (*%*)**			**.022**^b^**	
No care level	2 (5.1)	20 (6.0)		22 (5.9)
Care level 0	9 (23.1)	28 (8.4)		37 (10.0)
Care level 1	22 (56.4)	175 (52.7)		197 (53.1)
Care level 2	6 (15.4)	104 (31.3)		110 (29.6)
Care level 3	0 (0)	5 (1.5)		5 (1.3)

*Note.* SD: standard deviation, school education (Range 0 to 18 years), BSFC-s: Burden scale for family CGs – short version (Range 0 to 30), community health services (Range 0 to 13), NPI-Q: Neuropsychiatric Inventory Questionnaire (Range 0 to 12), ETAM: Erlanger test of Activities of daily living (Range 0 to 30), MMSE: Mini-Mental State Examination (Range 0 to 30)

^a^ t-test

^b^chi-square test

* The three cells “daughter−/son-in-law,” “other relatives,” and “friend/neighbor” were combined because these cells had an expected count of less than 5 in the chi-square test

** The cells “no care level” and “care level 0” as well as the cells “care level 2” and “care level 3” were combined because these cells had an expected count of less than 5 in the chi-square test

Bold printed *p*-values are statistically significant; *p* ≤ .05

### Intervention

The MAKS therapy consists of four components: motor skills (M), daily practical skills (A), cognitive skills (K), and social skills (S), and there is a manual for carrying out the group therapy [23]. The various components are performed daily in the same order and take about two hours. The therapy begins with a social attunement that lasts for about 10 min (e.g., a welcoming round). This is followed by exercises for sensorimotor activation (e.g., exercises to loosen up). This unit takes about 30 min and promotes general mobility, balance, as well as coarse and fine motor skills and sensory perception. This is followed by a break and then the cognitive activation, which also lasts about 30 min (e.g., remembering, recognizing). Participants are trained to develop skills such as logical thinking and speech comprehension. Finally, the daily practice activation (e.g., household activities) takes about 40 min. Coarse and fine motor skills are promoted, and procedural memory is stimulated [23]. In the intervention group, the MAKS therapy was conducted daily from Monday to Friday with all study participants present in the DCF. This resulted in a therapy dose of 1 to 5 treatment days per week [20]. In addition to the MAKS therapy, the CGs in the intervention group received up to three telephone calls from a trained counsellor with the goal to empower the caregivers by improving their skills in dealing with the home care [22].

### Measures

#### Mini-mental state examination (MMSE)

The MMSE measures different areas of cognitive functioning: orientation, registration, attention, arithmetic, memory, and language. The score to be achieved ranges from 0 to 30 points, with higher scores representing better cognitive performance. Scores of 23 or lower indicate dementia [24]. Reliability is α = .82 [25].

#### Montreal cognitive assessment (MoCa)

The MoCa is a psychometric test that is used to identify MCI. It consists of more difficult items than the MMSE. Scores of 0 to 30 can be achieved, with higher scores standing for better cognitive performance. Scores of 22 or lower indicate MCI. Reliability is α = .83 [26].

#### Resource utilization in dementia (RUD)

The RUD is the world’s most widely used questionnaire for collecting data on the use of resources by people with dementia. For the present study, the RUD was important for determining which community health services were being used. The community health services requested by the RUD included: nursing courses, a counseling center for relatives, a self-help group for relatives, home care services, care groups, “meals on wheels”, temporary institutional respite care, an outpatient nursing service, domestic help, and other services [27]. Possible answers were “yes” and “no”. Services that did not fit into any of the categories were summarized under “other services,” which included, for example, a medical call button or tandem holidays. With the information from “other services,” we expanded the list to include preventive care and non-drug treatments (e.g., physiotherapy, occupational therapy, or speech therapy).

#### Burden scale for family caregivers - short version (BSFC-s)

The BSFC-s comprises 10 items that are used to record the subjective burden on caregiving relatives. The 10 items are answered on a 4-point scale ranging from 0 (*strongly disagree*) to 3 (*strongly agree*). The higher the total score (0 to 30) on the BSFC-s, the stronger the subjective burden on the CG. Reliability is α = .92 [28].

#### Neuropsychiatric inventory questionnaire (NPI-Q)

The NPI-Q is a questionnaire for the evaluation of neuropsychiatric symptoms. The observer scoring scale is completed by an informal CG and includes 12 general categories of symptoms that are probed for presence/absence: delusions, hallucinations, agitation, depression/dysphoria, anxiety, elation/euphoria, apathy/indifference, disinhibition, irritability/lability, motor restlessness, nocturnal behavior, and appetite/eating. If the answer is “yes,” additional aspects can be investigated in this domain, but only the screening questions were administered in the present study [29].

#### Erlanger test of activities of daily living (ETAM)

The ETAM tests for the ability to perform instrumental activities of daily living. It is a performance test used to assess people with MCI or mild dementia. The test takes 19 to 35 min and consists of six items based on the International Classification of Functioning, Disability, and Health (ICF). Scores range from 0 to 30, with higher scores indicating better everyday practical skills. Reliability is α = .71 [30].

#### Care level

The care level in Germany is defined by the Medical Service of the Health insurances and depends on the need for care due to physical or mental disabilities and also determines which financial services the long-term care insurance will pay for. Trained experts from the medical services of health insurance companies (MDK) assess the need for care and distribute the care levels accordingly. The classifications range from a care level of 1 (*little impairment*, at least 1,5 h nursing time/ day) to a care level of 3 (*severe impairment*, at least 5 h nursing time/ day) [31]. An extra classification (care level 0) is given to people with dementia or mentally ill people who have impaired competence in instrumental activities of daily living (iADL) and Behavioral and Psychological Symptoms of Dementia (BPSD). This means limitations in at least two out of 13 areas, which include e.g. uncontrolled leaving of the living area; misjudging or causing dangerous situations; physically or verbally aggressive behavior; disturbance of the day-night rhythm [32].

#### Demographic data and care situation

DCF employees collected sociodemographic data (age, sex) and information about the care situation. CGs were asked to provide information about marital status, living situation, and educational level for both the CRs and the CGs. Information from the CGs about employment, living situation, and care situation (e.g. date of institutionalization or death) was recorded at each measurement point [20]. For a detailed description please also the study protocol [22].

#### Generation of sum scores ​​and differences

For the statistical calculations, the individual variables from the RUD were converted into sum scores. The final variable shows how many community health services were used in total. To account for any changes in the 6 months before our starting point, difference scores were calculated between the sum scores from our starting point (t6) and the measurement 6 months before (t0) (t_6_ - t_0_). Difference scores were calculated in the same way for the BSFC-s, the NPI, the care level, the MMSE, and the ETAM. Cox regression involves a time variable, which consist of days, months, or years. For this purpose, the variable “date of institutionalization” was recalculated as “months from the second measurement point (t_6_) to the transition to a nursing home”. Up to the middle of the month, the months were rounded down, and after the middle of the month, they were rounded up.

### Statistical analyses

To develop a multivariate predictive model of institutionalization of people with MCI, mild or moderate dementia visiting a DCF, a multivariate Cox regression was prepared. Therefore, the following approach was taken: For the descriptive statistics, t-tests and chi-square tests were calculated. To achieve meaningful results in the multivariate Cox regression, the number of variables should be limited. As a rule, the number of independent variables should not exceed 10% of the target events [33]. For the present study, this meant a limit of four independent variables for the multivariate model. To choose the most predictive variables, in a first step, 17 individual bivariate Cox regressions were calculated by using all the variables derived from the literature and a control variable:

1. age CR, age CG, educational level of CG, cohabitation, community health services use, subjective burden CG, cognitive skills CR, everyday practice skills CR, neuropsychiatric symptoms CR, and care level CR;
2. changes in the following variables across a 6-month time period (t6 - t0): CGs subjective burden, use of community health services, cognitive skills, everyday practical skills, neuropsychiatric symptoms, and care level
3. control variable: participation in the MAKS therapy/ telephone intervention.

For all Cox regressions, the inclusion method was used and institutionalization was the dependent variable. Since only categorical and metric variables can be used in the Cox regression, the ordinal variable “care level” has been converted into a categorical variable (no care level and care level 0 vs. care level 1, 2 and 3). To avoid redundancy in the multivariate model, the significant variables were tested for multicollinearity by performing a collinearity diagnosis. The requirement is met if all variance inflation factors (VIF) ​​are smaller than 5 [34]. The significant variables found in the bivariate analyses and the control variable “participation in the MAKS therapy (yes vs. no)” were included in the multivariate model to predict institutionalization. This was calculated by applying the multivariate Cox analysis with the inclusion method. For all analysis, an error probability (alpha) of less than 5% was the cutoff for statistical significance. The statistics software IBM SPSS Version 21 for Windows was used for all calculations.

## Results

The CGs mean age was 59.4 years, 73.6% were women and 53.4% were employed. The CRs mean age was 81.4 years and 62.3% were women. The majority of CRs in both groups (institutionalization and no institutionalization) had care level 1 (56% vs. 53%). In the case of institutionalization, 23% of the CRs had a care level of 0, whereas this was evident in 8% of CRs who were not institutionalized. For more information about the total sample as well as group comparisons of the two subgroups “institutionalization” and “no institutionalization,” see Table 1. The table is divided into information about the CGs and the CRs.

Table 1.

### Bivariate analysis

The results of the bivariate analyses are shown in Table 2. The significant predictors of institutionalization were: (1) cohabitation of CGs and CRs, (2) neuropsychiatric symptoms, and (3) care level.

**Table 2:** Bivariate Cox regressions

Variable	B	SE	Wald	p-Value	Exp(B)	95% Cl for Exp(B)	
						Lower	Upper
Age CR	.045	.025	3.169	.075	1.046	.995	1.099
Age CG	−.007	.014	.265	.607	.993	.966	1.020
Educational level of CG	.046	.050	.856	.355	1.047	.950	1.155
**Cohabitation**	**.884**	**.326**	**7.376**	**.007**	**2.421**	**1.279**	**4.583**
Community health services	.123	.116	1.126	.289	1.131	.901	1.419
Subjective burden CG	.024	.020	1.438	.230	1.024	.985	1.066
Cognitive skills CR	−.026	.026	.948	.330	.975	.925	1.026
Everyday practical skills CR	−.018	.021	.749	.387	.982	.942	1.023
**Neuropsychiatric symptoms CR**	**.190**	**.061**	**9.764**	**.002**	**1.210**	**1.074**	**1.363**
**Care level CR**	**.764**	**.356**	**4.607**	**.032**	**1.246**	**1.069**	**4.312**
Difference community health services	−.046	.147	.099	.753	.955	.716	1.273
Difference subjective burden of CG	.025	.034	.537	.464	1.025	.960	1.095
Difference cognitive disabilities	−.027	.041	.443	.506	.973	.898	1.054
Difference everyday practical skills	−0.27	.035	.575	.448	.974	.909	1.043
Difference neuropsychiatric symptoms	−.009	.088	.011	.916	.991	.824	1.177
Difference in care level	−1.022	1.243	.676	.411	.360	.031	4.116
MAKS therapy/telephone intervention	−.516	.347	2.211	.137	.597	.302	1.178

*Note.* B: regression coefficient, SE: standard error, Wald: Wald significance test, Exp (B): hazard ratio, Cl: confidence interval

Bold printed variables are statistically significant; p ≤ .05

### Multicollinearity

The collinearity diagnosis only showed VIF values ​​less than 5 (cohabitation: VIF = 1.025, neuropsychiatric symptoms: VIF = 1.021, care level: VIF = 1.002). Thus, all three predictors could be included in the multivariate analysis.

### Multivariate analysis

Table 3 shows the result of the multivariate Cox regression. The analysis shows that the risk of institutionalization increases: (1) when CRs and CGs do not live together (in the same house/apartment or in the same house but in separate apartments) (*p* = .005); (2) when the number of neuropsychiatric symptoms increases (*p* = .000); (3) when the CRs have a lower care level (no care level or a care level of 0 vs. care level 1 or 2 or 3) (*p* = .020).

**Table 3:** Multivariate Cox regression

Variable	B	SE	Wald	Sign.	Exp(B)	95% Cl for Exp(B)	
						Lower	Upper
**Cohabitation**	**.928**	**.327**	**8.055**	**.005**	**2.529**	**1.332**	**4.799**
**Neuropsychiatric symptoms (NPI-Q)**	**.218**	**.062**	**12.186**	**.000**	**1.243**	**1.000**	**1.405**
**Care level**	**.831**	**.356**	**5.442**	**.020**	**2.529**	**1.142**	**4.618**
MAKS therapy/ telephone intervention	−5.74	.348	2.724	.099	.563	.285	1.114

*Note.* B: regression coefficient, SE: standard error, Wald: Wald significance test, sign.: Significance, Exp (B): hazard ratio, Cl: confidence interval

Bold printed variables are statistically significant; p ≤ .05

## Discussion

The aim of the present study was to create a model for predicting institutionalization in people with MCI to moderate dementia who attend a DCF. Our results indicate that institutionalization is more likely (1) when the CRs showed more neuropsychiatric symptoms, (2) when the CRs and their CGs did not live together in the same house, or (3) when the care level of the CRs is low.

(1) When people with MCI to moderate dementia who attended a DCF had more neuropsychiatric symptoms (e.g. delusions, hallucinations, agitation, depression, anxiety, euphoria, apathy) they were more likely to be institutionalized. This predictor is particularly interesting because studies have already shown that neuropsychiatric symptoms can be influenced by therapy [20, 35]. This could be a starting point for therapeutic interventions to delay or prevent institutionalization.

(2) CRs who did not live with their CGs either in the same house or in the same house but with an extra apartment had an increased risk of institutionalization. This could be explained by various reasons: First, CRs who live with their CGs in the same house have a closer relationship. This makes it harder for both sides to consider institutionalization [36]. Second, when CGs and CRs do not live in the same house, CGs have to spend more time administering the care and traveling, which in turn fosters institutionalization [2]. There is no significant difference for institutionalization regarding CRs living in a relationship, which implies that the CR does not live alone. Therefore, to prevent institutionalization, it seems to be more important to live together with the CG than living together with someone. Although qualitative research shows, that institutionalization is a very complex and individual experience for both CGs and CRs [37–39] it seems to be particularly important to pay attention to those CRs where living together with the CG is not possible because they have a higher risk of institutionalization.

(3) Surprisingly, in this target group we found that the risk of institutionalization was higher when the level of care was lower (especially care level 0). Care level 0 was introduced subsequently for the target group of dementia and mentally ill patients, and their special needs, especially with regard to impaired competence in iADL and BPSD. Indirectly this could lead to the conclusion that the risk of institutionalization is higher if impaired competence in iADL and BPSD emerges. We hypothesize that impaired competence in iADL and the existence of BPSD is dominant in comparison to somatic need of care. This would be in line with our finding that a higher value in the NPI (which covers some areas of BPSD) leads to a higher risk of institutionalization. Generally, the interpretation of care levels is difficult as care level 0 opens up a new dimension compared to the other levels of care and therefore cannot necessarily be interpreted in a linear way.

The other predictors we tested, such as the age of the CRs, the age of the CGs, the level of education of the CGs, the subjective burden on the CGs, the use of community health services, and all variables including the changes across a 6-month period did not show a significant influence on institutionalization. The fact that we have focused on a special subgroup may have led to our results.

### Strengths of the study

The DCFs were distributed across Germany and differed regarding size and location (e.g. small and large DCFs, DCFs in urban and rural areas). Thus, considerable distortions in the sample could be avoided and generalizability of the results can be assumed. Furthermore, there is no research on institutionalization in people with MCI to moderate dementia who attend a DCF. The study highlights a specific group of people who differ in terms of the predictors of institutionalization from CRs who do not attend a DCF.

### Limitations and further research

First, we considered a relatively short period of time here, that is, change in the predictors across a 6-month period or the effect of certain variables on institutionalization during the next 6 months. It would be interesting to examine the relationships between variables across a longer period of time, such as Eska and colleagues did for a 4-year period [2]. Second, the focus in this study was on the earlier stages of dementia. Thus, severe dementia was not covered. Third, only CRs that have a CG were considered. Thus, CRs who do not have a CG could not be assessed. In addition, caregivers were only assessed with quantitative method. Qualitative data on reasons for institutionalization could generate new insights. Forth, the study is a secondary analysis of an intervention study. A part of the sample participated in MAKS therapy for 6 months, which had an impact on neuropsychiatric symptoms and everyday practical and cognitive abilities. The CGs received three telephone interventions. To control this influence we included the moderator variable “MAKS therapy and telephone intervention (yes vs. no)” in the multivariate Cox regression. To test the predictors independently of any intervention, future research should focus on samples in care as usual. Furthermore, the variable “frequency of attendance at DCF”, which could have an influence on institutionalization, was only collected at baseline and could therefore not be included in the analysis.

With regard to neuropsychiatric symptoms as the strongest predictor of institutionalization, it would be interesting to clarify which symptoms have the greatest impact. In a study by Okura and colleagues [40] the authors found that depression, delusions, and agitation as individual factors predict institutionalization.

## Conclusions

The present study contributes to the clarification of the predictors of institutionalization in people with MCI to moderate dementia who visit a DCF: Neuropsychiatric symptoms, which are the strongest predictor of institutionalization in this target group, are potentially influenceable. The literature shows that neuropsychiatric symptoms can be influenced by non-pharmacological interventions [20, 35]. This could be a starting point to “prevent” the institutionalization. Furthermore, when the CRs and their CG live together, this situation is a “noticeable protection” against institutionalization. If living together is not possible, counseling services should be used to be informed about support services. In addition, our results showed that, the risk of institutionalization becomes relevant with the care level 0, when limited competence in completing one’s daily routine tasks appears. This shows that the vulnerable phase of institutionalization in this specific group is earlier than often hypothesized [21]. CGs whose CR is experiencing those limitations for the first time in the course of the disease need specific counseling in order to cope with the situation and prevent institutionalization.

## Data Availability

The research group intends to publish data generated from this study in open-access, peer-reviewed journals. The datasets used and/or analysed during the current study will be available from the study coordinator (elmar.graessel@uk-erlangen.de) on reasonable request.

## Abbreviations

MCI: *Mild cognitive impairment*
CG: *Caregiver*
CR: *Care receiver*
DCF: *Day care facility*
MMSE: *Mini-Mental State Examination*
MoCa: *Montreal Cognitive Assessment*
RUD: *Resource Utilization in Dementia*
BSFC-s: *Burden Scale for Family Caregivers – short version*
NPI-Q: *Neuropsychiatric Inventory Questionnaire*
ETAM: *Erlanger Test of Activities of Daily Living*
iADL: *Instrumental Activities of Daily Living*
BPSD: *Behavioral and Psychological Symptoms of Dementia*
